# Understanding the Role of the Unfolded Protein Response Sensor IRE1 in the Biology of Antigen Presenting Cells

**DOI:** 10.3390/cells8121563

**Published:** 2019-12-04

**Authors:** Felipe Flores-Santibáñez, Bernardita Medel, José Ignacio Bernales, Fabiola Osorio

**Affiliations:** Laboratory of Immunology and Cellular Stress, Program of Immunology, Institute of Biomedical Sciences, Faculty of Medicine, University of Chile, Santiago 8380453, Chile; felipeflores.uchile@gmail.com (F.F.-S.); bernardita.medel@ug.uchile.cl (B.M.); jose.bernales@ug.uchile.cl (J.I.B.)

**Keywords:** unfolded protein response, immunity, antigen presenting cell, inflammation, infection

## Abstract

The unfolded protein response (UPR) is an adaptive response that maintains the fidelity of the cellular proteome in conditions that subvert the folding capacity of the cell, such as those noticed in infection and inflammatory contexts. In immunity, the UPR sensor IRE1 (Inositol-requiring enzyme 1-alpha) has emerged as a critical regulator of the homeostasis of antigen presenting cells (APCs). In the past few years, it has become clear that IRE1 plays canonical and non-canonical roles in APCs, many of which intersect with key features of these cells, including the initiation of inflammation, antibody production, and antigen presentation. The aims of the present review are to provide recent insights on the mechanisms by which IRE1 regulates the diversity of APC functions and to highlight its relevance in the coordination of innate and adaptive immunity.

## 1. Introduction

Antigen presenting cells (APCs)—composed of dendritic cells (DCs), B cells, and macrophages—are chief sentinels of the immune system and major cellular sensors of infection and tissue malfunction. These cells detect cell-extrinsic or cell-intrinsic inflammatory cues and respond by coordinating key immunological processes. Macrophages are tissue-resident phagocytes with multiple functions, including the production of inflammatory mediators that induce immune cell mobilization to the affected area (M1-polarization) or production of growth factors to promote tissue repair (M2-polarization) [1,2]. Furthermore, macrophages also promote the activation of T cells at the local site [1,2]. In contrast, DCs capture local antigens (Ags) and migrate to draining lymph nodes to present these Ags in the context of the major histocompatibility complex (MHC) to cognate naïve T cells, initiating long-term immunity against the insult [3,4]. Concomitantly B cells, which are key producers of antibodies, internalize and present Ags to T cells in lymph nodes, which in turn provide the necessary signals to differentiate into high-affinity antibody-producing plasma cells and memory B cell populations [5]. Altogether, the division of labor between macrophages, DCs and B cells allow eradication of the noxious threat and restoration of tissue homeostasis. In this context, it is known that APCs increase the protein synthesis rate in the endoplasmic reticulum (ER) upon activation by environmental cues [6,7,8]. This process facilitates the synthesis of co-stimulatory molecules, chemokines and cytokines, and antibodies [9,10,11,12]. The signal transduction pathway that monitors the protein folding capacity of the ER is termed the ‘unfolded protein response’ (UPR) and constitutes a major mechanism safeguarding the fidelity of the cellular proteome [13,14]. On the one hand, activation of the UPR modulates key steps of the secretory pathway, reprogramming the protein synthesis capacity of the cell [13,14], but on the other hand, in conditions of irrevocable ER stress, the UPR can also execute cell death in a process termed ‘maladaptive UPR’ [15].

Despite its canonical role in protein homeostasis, the relevance of the UPR in the biology of DCs, B cells, and macrophages stretches far beyond its classical function [16,17,18]. Key UPR members, such as the sensor IRE1 (inositol requiring enzyme 1 alpha), have proven to be essential in supporting development and function of DCs and plasma cells [19,20,21,22] and in promoting immunity to infection [23,24]. Nevertheless, even though the field has made enormous progress in recent years, the precise mechanisms by which IRE1 and the UPR regulate the homeostasis of APCs are not fully elucidated, as divergent outcomes have been reported depending on the subtype of APC involved, or on whether the inflammatory context elicits adaptive or maladaptive UPR [17,25,26]. Thus, depending on the physiological or pathophysiological context, the UPR can be beneficial or detrimental to APC function. These findings indicate that the field is evolving and we still have a long way to go before obtaining a comprehensive understanding of the role of the UPR in the control of immunity. In this review, we discuss key findings on the role of the UPR with a focus on the sensor IRE1 in the homeostasis and function of APCs.

## 2. The Unfolded Protein Response

**Figure 1:**
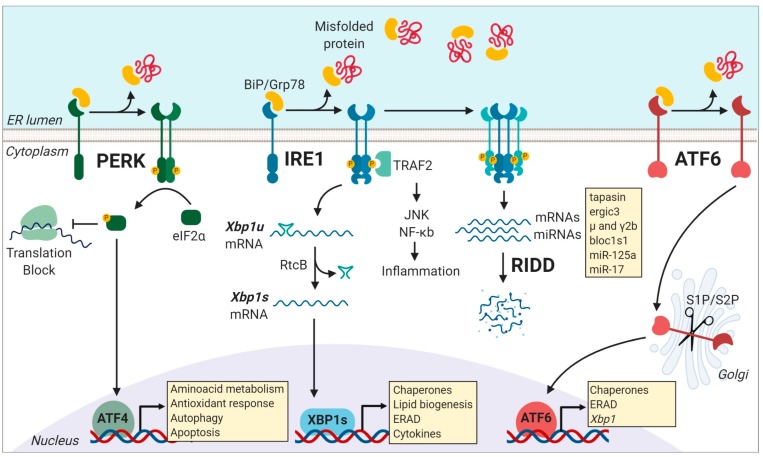
Activation of the three unfolded protein response (UPR) pathways is initiated by misfolded protein accumulation in the endoplasmic reticulum (ER). Phosphorylation of the eukaryotic translation initiation factor 2α (eIF2α) is dependent of the PKR-like kinase (PERK). This process inhibits ribosome assembly, which causes a translational block allowing the cell to cope with temporary ER-stress. However, ATF4 escapes this translation inhibition under conditions of stress and induces the transcription of genes related to cell survival including those involved in compensatory autophagy. Activation of the endoribonuclease domain of IRE1 is caused by the dissociation of the binding immunoglobulin protein (BiP) from the luminal domain of IRE1, causing the non-conventional splicing of the unspliced form of the X-box binding protein 1 (*xbp1u*) mRNA to produce *xbp1s* mRNA, which encodes the potent transcriptional activator, XBP1s. Among the various targets of XBP1s are genes encoding for chaperones, genes that assist in the degradation of misfolded proteins via ER-associated degradation (ERAD), lipid biogenesis, and cytokine production. Under conditions of chronical stress, IRE1 is hyper-activated, and it cleaves additional RNAs, such as mRNAs and miRNAs, through a process called Regulated IRE1 dependent decay (RIDD). After BiP dissociation from ATF6 during ER stress, ATF6 travels to the Golgi compartment, where it is processed by the S1P/S2P enzymes. The processed ATF6 fragment functions as a transcription factor that enhances protein folding at the ER level and also promote the expression of target genes that assist in degradation processes, including ERAD. Figure created with Biorender.com.

The ER plays a pivotal role in synthesis, folding, and maturation of up to 30% of proteins in the cell [14]. This organelle is also the major reservoir of intracellular Ca^2+^ and a hub for lipid biosynthesis for cellular membranes [13]. Changes in physiological or environmental demand lead to rapid increases in the protein-folding rate in the ER. As mentioned above, the development of an immune response requires synthesis of a broad range of proteins, including inflammatory mediators, costimulatory molecules, and effector molecules, such as antibodies. However, if the protein folding capacity of the cell is not synchronized with the protein folding demand, misfolded proteins are accumulated in the lumen of the organelle causing ‘ER stress’ [27]. Additionally, many conditions that are intrinsic to the inflammatory milieu can disrupt the cell folding machinery, such as hypoxia, nutrient deprivation, and the presence of reactive oxygen species (ROS), which induces ER-stress [16,28]. ER stress in turn activates the UPR, an adaptive cellular response controlled by three ER-resident sensors: PERK (protein kinase R-like ER kinase), ATF6 (activating transcription factor 6 alpha), and IRE1 [27] (Figure 1). In resting conditions, the UPR sensors are held in an inactive state through their association with the chaperone BiP/Grp78 [29]. Upon ER stress, BiP dissociates from the sensors due to preferential binding to misfolded proteins, allowing for activation of the UPR [14,30]. In broad terms, the UPR controls the expression of genes that improve the protein folding capacity of the cell and restore ER homeostasis [14]. However, in conditions of irrevocable ER stress, the UPR can directly execute cell death [15]. As such, the mechanisms accounting for the switch from adaptive to maladaptive UPR are matters of extensive research. Remarkably, UPR activation has emerged as a hallmark of several diseases, including inflammatory bowel disease, arthritis, neurodegenerative diseases, diabetes mellitus, stroke, and cancer among others [15,16,17], and small molecules targeting UPR components have shown protective effects in various models of neurodegeneration, metabolic disorders, and cancer [31,32].

PERK is a type I transmembrane kinase that under ER stress oligomerizes and auto *trans*-autophosphorylates and globally inhibits protein translation via the phosphorylation of eif2α (eukaryotic translation initiator factor-2) [27]. At the same time, P-eif2α promotes selective translation of proteins that control cell survival, ER homeostasis, and antioxidant response modules, such as the transcription factor ATF4 [33] (Figure 1). ATF4 directs the expression of the pro-apoptotic factor CHOP (C/EBP homologous protein) and GADD34 (growth arrest and DNA damage-inducible protein 34), which is a negative regulator of eif2α phosphorylation [13,14]. Remarkably, P-eif2α is not only controlled by PERK as it can also be activated by additional kinases that control viral infection, nutrient deprivation, or heme deficiency, which represents a point of convergence of different stress pathways in a process referred to as the “integrated stress response” [34]. In immunity, selective translation mediated by P-eIF2α influence the repertoire of peptides presented in MHC-I molecules [35]. Activation of P-eIF2α allows selective translation of mRNAs bearing upstream open reading frames (uORFs) in their 5′ untranslated regions (5′ UTR), which serve as novel Ags for MHC-I presentation [35].

IRE1 is the most conserved UPR sensor and the most studied in the immune system [16,17,36]. IRE1 is a type I transmembrane protein with a serine/threonine kinase domain and an endoribonuclease (RNase) domain [13,14]. Upon activation, IRE1 dimerizes and auto *trans*-phosphorylates, activating its RNase domain, which catalyzes the excision of a 26-nt intron within the *Xbp1* (X-box binding protein 1) mRNA sequence [37,38,39,40]. This unconventional splicing event is completed by the protein RtcB, which ligates the *Xbp1* spliced mRNA, allowing translation of the active transcription factor XBP1s [41,42,43]. XBP1s is a master regulator of genes involved in lipid biosynthesis, protein folding, ER-associated degradation (ERAD), and ER biogenesis [44,45]. Furthermore, in poorly-defined conditions of chronic ER stress or in certain secretory cell types deficient in XBP1s, IRE1 is hyper activated and expands its substrate repertoire by cleaving additional ER-localized RNAs and microRNAs (miRNAs) through a process termed ‘Regulated IRE1 Dependent Decay’ or RIDD [46,47] (Figure 1). RIDD was originally proposed as a mechanism aiming to alleviate the protein folding load during ER stress and its substrates bear a consensus element accompanied by a stem-loop structure, which is also present in the *Xbp1* unspliced mRNA [48]. RIDD is associated with key biological functions related to inflammation, metabolism, and survival [49], and reported substrates of the enzyme include insulin, pro/anti-apoptotic miRNAs, and members of the antigen presentation machinery such as tapasin, among others [21,50,51,52]. Within APC subtypes, RIDD has emerged as a key regulator of the homeostasis of plasma cells and type 1 conventional DCs (cDC1s) [21,22,53] (see below). As such, IRE1 RNase is a regulator of protein homeostasis via two distinct pathways: (1) Transcriptional activation and (2) RNA decay. The molecular mechanisms by which IRE1 RNase co-opts for XBP1s or RIDD are current matters of intense research. Reported evidence indicates that the switch between XBP1 splicing and RIDD occurs with different kinetics [54], and it is influenced by the oligomerization status of IRE1 [54,55]. Furthermore, recent work has identified key residues in the IRE1 kinase domain that are required for selective RIDD activation [56]. 

In addition, the kinase domain of IRE1 can also couple ER stress to inflammation [57,58]. IRE1 kinase activate JNK (c-Jun N-terminal kinase), TRAF2 (TNF receptor-associated factor 2), and NF-kB signaling modules [59,60], which can directly initiate inflammatory responses. Remarkably, IRE1 kinase activity contributes to the development and function of Paneth cells and the establishment of intestinal homeostasis [58,61].

However, it has been demonstrated that the levels of XBP1s are critical to dictate survival versus cell death [62]. In conditions of persistent ER stress, XBP1s promote transcription of the cell-death associated factor KLF9 [62], which possess a low affinity binding site for XBP1s and therefore requires substantial accumulation of XBP1s for activation [62], providing a mechanism linking the IRE1/XBP1 axis with the transition to maladaptive UPR.

ATF6 is an ER transmembrane protein that contains a bZIP transcription factor on its cytosolic domain. Upon ER stress, ATF6 is translocated to the Golgi apparatus, where it is cleaved by site-1 and site-2 proteases, resulting in the release of a transcription factor that controls the expression of chaperones, ER-Associated protein degradation (ERAD) components, and proteins involved in lipid biogenesis [13,27] (Figure 1). Notably, transcriptional targets of ATF6 include the transcription factor XBP1, and thus, ATF6 is recognized as a regulator of the IRE1/XBP1s axis [37,38,63]. In immunity, it has been reported that ATF6 is activated upon recognition of bacterial products and synergize for the production of proinflammatory cytokines [23,64,65], presumably via NF-kB activation [66,67]. In addition, considering that ATF6 controls the expression of members of the ER quality control machinery [68,69] and the fact that some components of this process directly influence antigen processing [18], it is plausible that ATF6 regulates antigen presentation by APCs. However, this is an aspect that remains to be formally demonstrated. Additionally, in light of the cross-talk between ATF6 and XBP1, the former transcription factor may also indirectly regulate the immunological functions of XBP1. Furthermore, it remains to be determined whether the ATF6-dependent downregulation of XBP1 is sufficient to trigger the activation of RIDD, which may also have an impact in fine-tuning immune responses. In this line, future studies are required to reveal the functional relevance of ATF6 axis and the ATF6-XBP1 cross-talk in immunity.

Finally, beyond the dedicated tasks operated by each branch of the UPR, cooperative interactions between the UPR transducers has also been reported. XBP1 can form heterodimers with ATF6, which regulates an additional core of target genes involved in protein homeostasis [68,69]. 

Moreover, recent evidence indicates that the UPR is not only initiated by the accumulation of misfolded proteins, but it can also be activated by additional sources of stress. For instance, IRE1 and ATF6 are sensitive to lipid metabolism and can be activated upon changes in membrane lipid composition, regulating gene expression [55,70,71,72,73]. This evidence indicates that the UPR is a complex network of signaling pathways that sense perturbations affecting ER homeostasis, including protein misfolding and lipid bilayer stress.

## 3. Control of Ag Presentation by IRE1

While macrophages, DCs, and B cells differ in ontogeny and perform non-overlapping functions in the immune system, all these lineages can present Ag to T cells to elicit long-term immunity. Different routes of Ag presentation lead to the activation of CD8^+^ and CD4^+^ T cells, respectively. For initiation of CD8^+^ T cell immunity, Ags of cytosolic origin are processed into peptides by the proteasome [74]. These peptides enter the ER via the transporter associated with Ag processing (TAP), and are edited and loaded into MHC-I molecules by the peptide loading complex, which is composed of TAP and the chaperones tapasin, calreticulin, and ERp57 [74,75]. The MHC-I/peptide complex then enters the secretory pathway to reach destination to the plasma membrane [75]. For Ag presentation to CD4^+^ T cells, Ag is phagocytosed and directed to early endosomes, which mature into late endosomes that recruits proteolytic enzymes (e.g., cathepsins), chaperones, and MHC-II molecules required for processing and loading of Ags into MHC-II molecules [76]. Interestingly, in a variation of the classical MHC-I pathway, certain DC subtypes (in particular cDC1s, see below) can divert exogenous Ags to the MHC-I Ag presentation route through a process referred to as ‘cross-presentation’, which is a prominent immune strategy to prime CD8^+^ T cells against tumors and virally-infected cells [77]. 

In this context, IRE1 has been shown to control several steps of the Ag presentation route. While XBP1s were originally described as a transcriptional regulator of MHC Class II expression [78,79], XBP1 deficient DCs were not impaired in presenting Ags to CD4^+^ T cells in steady state [21]. However, this evidence does not exclude the possibility that the transcription factor may play a role in CD4^+^ T cell priming in pathological or inflammatory settings. On the other hand, prominent findings connect the IRE1/XBP1s axis with Ag presentation in MHC class I (reviewed in [18]). Reported transcriptional targets of XBP1s include MHC-I folding genes (calnexin); antigen processing genes (proteasomal subunits Rpn1/Rpn2) and MHC-I/Peptide binding genes (TAP, calnexin, and members of the peptide loading complex: Calreticulin and ERp57, which is a shared target with ATF6) [18,69,80]. It is important to consider that most evidence emerged from studies in cell lines or non-professional APCs and further studies are required to obtain a clear view of the role of the UPR in Ag processing routes in APCs. However, tapasin has already been identified as a RIDD substrate in cDC1s, and on a functional level, XBP1 deficiency curtails the cross-presentation abilities of cDC1s, demonstrating the key role of the pathway in the functionality of this cell subtype [21] (see below). 

## 4. IRE1 in the Regulation of APC Biology 

**Figure 2:**
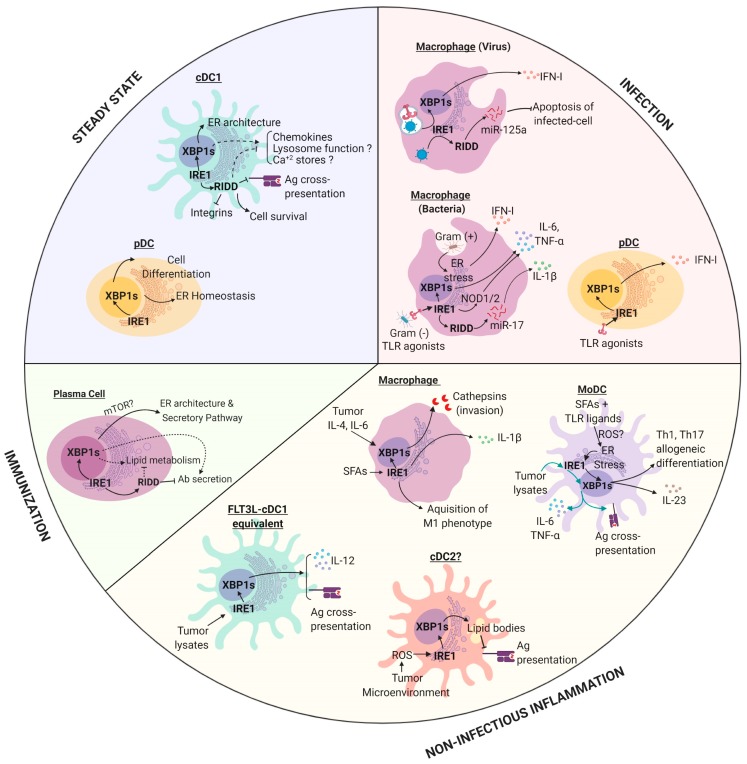
The role of IRE1 in antigen presenting cells (APCs) in physiology and pathophysiology. IRE1 is activated by APCs in different contexts and the outputs of this response are both cell-type and context dependent. Dashed lines: Mechanism remains to be fully elucidated. Abbreviations: cDC1, conventional Dendritic Cells of type 1; cDC2, conventional Dendritic Cells type of 2; pDC, plasmocytoid Dendritic Cell; MoDC, Monocyte derived Dendritic Cell; IRE1, Inositol-requiring Enzyme 1 alpha; XBP1, X-box Binding Protein 1; RIDD, Regulated IRE1-dependent decay; SFAs, Saturated Fatty Acids; TLR, toll like receptor. All RIDD-related responses were studied in models where XBP1s was ablated. Figure created with Biorender.com.

In addition to the common features controlled by IRE1 in Ag presentation, the UPR sensor also played dedicated functions in APCs depending on the cell subtype (Figure 2):

### 4.1. Macrophages

As cellular sensors of the immune system, tissue resident macrophages are located across several tissues. These cells capture microbes, particles and dying cells and initiate immunity via production of chemokines and cytokines, additional factors, and through local activation of T cells [81,82]. Tissue resident macrophages can have an embryonic origin (yolk sac, and fetal liver), or can be differentiated from bone marrow-derived monocytes depending on the tissue of residency [83]. In infection, high numbers of macrophages are differentiated de-novo from BM-derived monocytes, which emerged to reinforce innate immunity at the infected site. Mice bearing XBP1 deletion in hematopoietic precursors showed normal macrophage frequency, indicating that the IRE1/XBP1s axis did not control the development of the lineage [84]. However, substantial evidence demonstrated a key role of IRE1 in the control of innate immunity to infection. Activation with viral agonists in presence of pharmacological ER stressors synergized for the production of type I Interferons (IFN-I) in macrophages, which has shown to depend on XBP1s activity [85]. Furthermore, XBP1 deficient macrophages displayed impaired anti-viral resistance due to the inability to induce apoptosis, which is a key step limiting viral spreading [52]. XBP1 KO macrophages were resistant to the intrinsic pathway of apoptosis upon viral infection, via a mechanism depending on IRE1-mediated cleavage of the proapoptotic miRNA-125a, which targeted the anti-apoptotic proteins Bcl-xl and Mcl-1 [52]. As such, data indicated that IRE1 RNase played a pro-survival role in viral infections by promoting resistance to apoptosis via a mechanism independent of IFN-I production and future studies are required to unveil the relevance of the process in vivo. Furthermore, small mRNA products generated by RIDD in non-infectious conditions can also lead to IFN-I synthesis in specific contexts. It has been reported that the activation of RIDD in macrophages deficient of the SKIV2L RNA exosome resulted in enhanced IFN-I production, suggesting that, in absence of quality controls responsible to degrade self RNAs, endogenous RIDD substrates can also initiate immunity [86]. 

Macrophages recognized pathogens through the so-called PRRs, which are innate receptors that recognized pathogen-associated molecular patterns and that signal for the activation of immune-related genes [87]. The archetypical family of PRRs is the toll-like receptor (TLR) family, which recognized different classes of microbes [87]. Remarkably, upon bacterial infection, TLR signaling activated the IRE1/XBP1s axis, which contributed to the optimal production of the proinflammatory cytokines IL-6, TNF, and pro-IL1β and it was required to eradicate bacteria [23]. On a mechanistic level, XBP1s directly binds to the promoter regions of IL-6 and TNF [23]. The relevance of XBP1 in the control of infection was underscored in a model of *Francisella tularensis* infection, where selective deletion of XBP1 in macrophages resulted in enhanced pathogen burden [23]. Remarkably, upon TLR ligation, ER stress also licensed macrophages to produce mature IL-1β though a pathway dependent on Caspase-8 and TRIF, suggesting a direct link between ER stress and the inflammasome [88]. Accordingly, RIDD was also reported to activate the inflammasome via degradation of miR-17, which was a destabilizer of the NLRP3 inflammasome activator TXNIP (thioredoxin-interacting protein), and thus, its degradation caused IL-1β secretion [89].

Moreover, activation of IRE1 kinase domain initiated inflammatory responses in macrophages [57]. IRE1 kinase directly activated TRAF2 signaling modules, which elicited activation of the PRRs NOD1/2 (nucleic-binding oligomerization domain-like receptors 1/2) for induction of IL-6 during *Brucella abortus* infection [57]. Activation of this pathway resulted in a strong proinflammatory response that contributed to abortion induced by *B. abortus* infection [57]. 

In infections with gram-positive bacteria, it was reported that *Listeria innocua* induced the UPR for initiation of autophagy and IFN-I production in macrophages [24]. Interestingly, IRE1 and PERK were recruited to pathogen-containing autophagosomes, demonstrating a direct link between autophagy and the sequestration of stressed ER membranes [24]. On a functional level, PERK deletion in macrophages resulted in enhanced infection with *Listeria monocytogenes*, demonstrating a key role of the UPR sensor in the control of innate immunity to the pathogen.

Notably, IRE1 could also modulate the function of macrophages in non-infectious contexts. Saturated fatty acids (SFAs), which were associated with tissue inflammation, obesity, and diabetes, induced ER stress and IRE1 signaling in these cells [72]. SFAs induced inflammasome activation and IL-1β secretion by a mechanism dependent on IRE1 RNase activity [72]. Furthermore, IRE1 activation was also noticed in adipose tissue macrophages in models of high fat diet [90]. In these settings, IRE1 coordinated the acquisition of a proinflammatory profile in macrophages (termed ‘M1’), which contributed to metabolic inflammation and obesity. In fact, selective IRE1 deletion in macrophages had marked effects in preventing high fat diet-induced obesity, hyperlipidemia, and insulin resistance [90]. As such, IRE1 emerged as the critical switch controlling macrophage polarization into inflammatory and anti-inflammatory profiles. 

Finally, many studies have also demonstrated a relevant role of the UPR in cancer-associated immune cells and the topic has been extensively reviewed elsewhere [26]. It has been reported that conditioned medium derived from tumor cells undergoing ER stress, can induce ER stress in macrophages via a process referred to as ‘transmissible ER stress’ [91]. Transmissible ER stress induced the activation of XBP1s, BiP, CHOP, and amplification of a proinflammatory phenotype [91]. Finally, sensing of the tumor-associated cytokines IL-4 and IL-6 by macrophages induced IRE1/XBP1s activity and increases the secretion of proteins that promote cancer cell invasion such as cathepsins [92].

### 4.2. Monocyte-Derived DCs (MoDCs)

As previously noticed in macrophages, monocytes also differentiate into ‘DC-like cells’ upon inflammation. These cells are referred to as ‘monocyte-derived DCs’ or MoDCs [93,94]. MoDCs display potent anti-microbial functions and share several features with conventional DCs, including enhanced cytokine production and proficient antigen presentation abilities, albeit with different efficiencies and using distinct transcriptional programs [94,95]. MoDCs was a prominent model of study of DCs in immunology because these cells could be generated in large numbers in in vitro cultures of bone marrow cells supplemented with a granulocyte–macrophage colony-stimulating factor (GM-CSF) [96]. However, in mice, these cultures also contained large numbers of macrophages, which complicated clear distinction between lineages [97]. However, the UPR and in particular IRE1, was reported to play relevant roles in this system, which has been extended to in vivo models of disease. Synthesis of IL-23, a relevant cytokine driving psoriasis and autoimmune diseases such as rheumatoid arthritis [98], was controlled by the IRE1/XBP1s pathway in MoDCs from human and mice origin [99]. IL-23 was also produced in high levels in murine MoDCs activated with TLR ligands plus pharmacological inducers of ER stress, via a process dependent on CHOP [100]. Furthermore, stimulation of MoDCs in the presence of TLR ligands plus fatty acids triggered an exacerbated UPR, which augmented the production of IL-23 and IL-6 [101]. Fatty acids induced metabolic adaptations in MoDCs that prevented glycolysis and induced XBP1s, CHOP, and ATF4, which promoted IL-23 production through a mechanism dependent on mitochondrial ROS [101]. In vivo, XBP1 deletion in DCs alleviated a model of psoriatic skin inflammation, which was IL-23 dependent [101]. Furthermore, human MoDCs infected with *Chlamydia trachomatis* synthetized IFN-I via a mechanism involving TLR4-IRE1 and PERK [102]. From a functional perspective, inhibition of XBP1s curtailed the capacity of human MoDCs to stimulate allogeneic Th1 and Th17 differentiation [103]. As such, compelling evidence demonstrated that the UPR was an essential cellular point of convergence for amplification of cytokine production and T cell priming in myeloid cells. Interestingly, IRE1, via XBP1s, also controlled prostaglandin biosynthesis and pain [104], indicating that the UPR sensor regulated inflammatory processes beyond the regulation of cytokine/chemokine production. 

In cancer models, transmissible ER stress also promoted proinflammatory cytokine secretion in MoDCs, but intriguingly, it impaired the generation of surface MHC-I/peptide complexes and Ag cross-presentation, which facilitated tumor growth [105]. It remains be determined which UPR sensor was responsible for this immunosuppressive effect. However, a different set of experiments demonstrated that recognition of melanoma cell lysates potently activated the IRE1/XBP1s axis in MoDCs, which increased the production of IL-6 and TNF and promoted cross-presentation of tumor-associated Ags *in vitro* [106]. Inhibition of IRE1 RNase activity, either through pharmacological blockade or by genetic deletion of the RNase domain, resulted in reduced cross-presentation of melanoma-associated Ags [106]. This evidence indicated that activation of IRE1 RNase in MoDCs contributed to effective CD8^+^ T cell activation. Accordingly, enforced expression of XBP1s rendered MoDCs more effective as vaccines in a melanoma setting, by promoting Ag presentation to CD4^+^ and CD8^+^ T cells [107,108]. Altogether, evidence demonstrated that IRE1 is a key regulatory sensor that controls the activation of MoDCs for production of cytokines and additional mediators that regulate innate immunity, while it is also a relevant coordinator of Ag presentation.

### 4.3. pDCs 

DCs are a highly heterogeneous population of leukocytes found in all organs that develop from hematopoietic precursors and that can be divided as plasmacytoid and conventional DCs (pDCs and cDCs, respectively) [109]. pDCs are major producers of IFN-α upon viral infection [110] and show constitutive XBP1s in steady state [20]. XBP1 deficiency resulted in marked reduction in pDCs frequencies [20] and the remaining population displayed a poorly developed ER and increased apoptosis [20]. Notably, constitutive XBP1s expression was also noticed in human pDCs [111] and genetic deletion of XBP1 in mice or treatment with a pharmacological inhibitor of the IRE1 RNase domain in PBMCs reduced IFNα upon stimulation with TLR ligands [20,111].

### 4.4. cDCs

cDCs comprised two major subtypes: cDC1s and cDC2s, which displayed specialized immune functions [112]. cDC1s required the transcription factors IRF8 and BATF3 for development [113,114,115] and on a functional level, the lineage exceled in cross-presenting Ags derived from tumors and virally-infected cells to CD8^+^ T cells [77,116] (see below). Due to these features, cDC1s have become central targets of immunotherapy [117]. The cDC2 lineage was more heterogeneous and depended on the transcription factors IRF4, Notch2, and KLF4 for development [112]. cDC2 displayed proficient abilities to present Ags from extracellular bacteria, fungi, and parasites to CD4^+^ T cells [112,115,118].

XBP1 deletion in hematopoietic precursors resulted in lower numbers of cDC1s and cDC2s, demonstrating a role of the pathway in cDC development [20,84]. In regards to cDC subtypes, IRE1 has emerged as a key regulator of cDC1 homeostasis. These cells displayed spontaneous IRE1 RNase activity in steady state, featured noticeably across several lymphoid and non-lymphoid organs with the exception of the lung [21,22]. Furthermore, the enhanced IRE1 RNase activity observed in cDC1s was not accompanied with a canonical UPR, suggesting that the axis played selective roles in cDC1 function in the absence of ER stress [21]. Importantly, cDC1s were highly sensitive to perturbations in IRE1 signaling and activate RIDD upon XBP1 deletion [21]. RIDD activation in the context of XBP1 deficiency was a hallmark of a selective group of cells that included cDC1s, plasma cells, and hepatocytes and the mechanism underlying the process has not been fully elucidated [21,53,119]. XBP1 KO cDC1s changed the transcriptome and showed reduced expression of transcripts that included regulators of lysosomal maturation (*lamp1, bloc1s1*), Ag processing (proteasomal subunits *rpn1/2*), MHC-peptide loading (*tapasin*, *h2-dm*), vesicular trafficking (*ergic3*), ER sensors of Ca^2+^ depletion (*stim1/2*), chemokines (*ccl22*), integrins (*cd18*), and DC receptors (*langerin*), among others [21]. As such, the IRE1/XBP1s axis may regulate several aspects of cDC1 homeostasis, but the precise contributions of each of these processes remains to be determined. Remarkably, although XBP1 KO cDC1s have no overall defects in protein secretion, these cells displayed an aberrant ER morphology typified by disorganized cisternae [21]. On a functional level, XBP1 deficiency rendered cDC1s unable to cross-present cell-associated Ags, which was a process attributed to RIDD [21]. While the mechanisms linking IRE1 with the cross-presentation pathway were not clearly elucidated, members related to the MHC-I/cross-presentation pathway such as *tapasin* and *ergic3* were identified as RIDD substrates [21].

Beyond Ag cross-presentation, IRE1 RNase also regulated the survival of cDC1s in mucosal tissues [22]. XBP1 deficiency resulted in cell death in cDC1s that resided in the lung, but the survival of the same subset in the intestine [22]. This differential effect was due to the selective ability of intestinal XBP1 KO cDC1s to shut down protein synthesis and activate RIDD, which acted as a pro-survival mechanism [22]. However, the precise mechanism remains to be determined. Altogether, the data indicated that activation of IRE1/XBP1s axis was intrinsically associated with the cDC1 lineage and can be added to the growing list of regulators of tissue residency and function of the subset.

However, in cDC2s, XBP1 deficiency did not lead to phenotypical or transcriptomic changes [21]. While no studies have formally addressed the role of the IRE1/XBP1s axis in bona fide cDC2s, some findings associated a detrimental role for the transcription factor in the function of the lineage. In ovarian cancer models, an aggressive tumor model that predominantly recruited cDC2s/MoDCs in mice and humans [25,120], XBP1s activation in tumor-associated DCs curtailed the abilities of these cells to prime antitumor T cells, resulting in exacerbated tumor growth [25]. It is proposed that sustained XBP1s activation, triggered by the tumor microenvironment, provoked maladaptive UPR and aberrant accumulation of lipid droplets in cDC2s, which inhibited antigen presentation [25]. At present, it is unclear what dictated the functional or dysfunctional role of IRE1 in DC subsets. Exhaustive studies aiming to carefully dissect the role of IRE1 in the cDC subset in physiology and pathology will provide valuable knowledge on the matter.

cDCs can be partially recapitulated in in vitro cultures of bone marrow cells containing FMS-like tyrosine kinase 3 ligand FLT3L [121]. In these cultures, inhibition of IRE1 RNase resulted in reduced cross-presentation of tumor-associated Ags in vitro and decreased the production of proinflammatory cytokines in response to exposure to tumor cell lysates [106]. IRE1 RNase blockade in cDC1 equivalents from FLT3-L-containing cultures resulted in lower expressions of surface MHC Class I/ peptide complexes and reduced IL-12 production upon recognition of tumor cell lysates, which accompanied the reduced cross-presentation abilities [106]. Accordingly, overexpression of XBP1s in a vaccination setting enhanced the cross-presentation abilities of cDC1s and promoted tumor elimination in a Batf3-dependent manner [94].

### 4.5. B Cells

Early studies using rag2^-/-^ blastocysts, reconstituted with XBP1^-/-^ embryonic stem cells, showed a role for XBP1 in plasma cell development [19]. XBP1s occur physiologically during plasma cell differentiation [19] and was independent of Ig synthesis [122,123]. In plasma cells, XBP1s was activated downstream of the transcription factor Blimp1 and expanded the secretory apparatus promoting increases in ER, lysosomal, and mitochondrial content [11]. While XBP1 deficient B cells displayed normal protein disulfide formation, and normal ER exit of proteins that enter the secretory pathway [124], these cells synthetized lower levels of IgG and IgA [12]. Furthermore, evidence demonstrated that XBP1s regulates late events of plasma cell differentiation, but it does not affect memory B cell formation [123,125]. In fact, mice bearing selective deletion of XBP1 in B cells generated low amounts of autoantibodies and are protected in a model of lupus [125]. Additional aspects regulated by the IRE1/XBP1 axis in B cells included terminal protein glycosylation and lipid synthesis [53,124]. Furthermore, as seen with cDC1s, deletion of XBP1s in plasma cells also resulted in RIDD activation, which in turn controlled the levels of mRNAs encoding for the µs and γ2b heavy chains [53]. On a functional level, RIDD attenuated IgM responses upon vaccination [53]. A recent study identified that phosphorylation of IRE1 at S729 was required for RIDD activation [56]. In agreement with previous findings [53], S729A mice produced higher levels of IgM and IgG2b upon immunization [56]. Intriguingly, activation of mTOR was able to override the ER morphology and the decreased Ig synthesis noticed in XBP1 deficient B cells [126], supporting the notion that metabolic programs and the UPR are closely intertwined to coordinate the function of immune cells. Future studies are needed to determine the precise mechanism of this crosstalk. 

## 5. Emerging Roles of IRE1 in APC Biology

In recent years, enormous progress has been made in elucidating the mechanisms by which IRE1 controls protein homeostasis. Remarkably, many of these processes may also be relevant for the APC function in a field that remains largely unexplored. In one respect, XBP1s directly control the expression of Ca^+2^ sensors, which are required for Ag processing in DCs [127,128]. Furthermore, RIDD is known to control lysosomal positioning and homeostasis [129], which may be relevant in DC migration and Ag presentation, as both processes require proper lysosomal function [130,131]. Moreover, novel roles for IRE1 have been reported recently, which are independent of the UPR, but that may also impinge on the APC biology. For instance, IRE1 is a novel regulator of cell migration, through a mechanism that involves interaction with filamin A [132], but it remains to be determined whether this process is relevant for cell migration to secondary lymph nodes by cDCs or not. In addition, transcriptomic analysis has also identified chemokines as direct targets of the IRE1 [21], but the in vivo relevance of the process has not been elucidated yet.

## 6. Conclusions

We are just beginning to appreciate the multiple functions of IRE1 in the variety of functions of APCs. To date, the overall picture is far from complete. As such, a major long-term goal is to integrate current knowledge with novel insights on the role of IRE1 in APC biology for generating a comprehensive theoretical framework that leads to translational applications.

## Abbreviations

APC	Antigen presenting cell
Ag	Antigen
ATF6	Activating transcription factor 6
IRE1	Inositol-requiring enzyme 1 alpha
PERK	PKR-like endoplasmic reticulum kinase
RIDD	Regulated IRE1-dependent decay
ATF4	Activating transcription factor 4
ER	Endoplasmic reticulum
ERAD	ER-associated protein degradation
DC	Dendritic cell
cDC	Conventional DC
pDC	Plasmacytoid DC
Mo-DC	Monocyte derived DC
MHC	Major histocompatibility complex
ERGIC	ER-Golgi intermediate compartment
PLC	Peptide loading complex
UPR	Unfolded protein response
TAP	Transporter associated with antigen processing
XBP1	X-box binding protein 1
CHOP	C/EBP homologous protein
GADD34	Growth arrest and DNA damage-inducible protein 34
